# Support for Health Insurance Coverage for Legal Abortion in the United States

**DOI:** 10.3390/ijerph19010433

**Published:** 2021-12-31

**Authors:** Charley Henderson, Philip Q. Yang

**Affiliations:** Sociology Program, Texas Woman’s University, Denton, TX 76204, USA; chenderson15@twu.edu

**Keywords:** health insurance coverage, legal abortion, the United States, General Social Survey

## Abstract

The use of health insurance to cover legal abortion is a controversial issue on which Americans are sharply divided. Currently, there is a lack of research on this issue as data became available only recently. Using data from the newly released General Social Survey in 2018, this study examines who is more or less likely to support health insurance coverage for legal abortion. The results show that the support and opposition were about evenly divided. The findings from the logistic regression analysis reveal that, holding other variables constant, Democrats, liberals, urban residents, the more educated, and the older were more likely to support health insurance coverage for legal abortion while women, Southerners, Christians, the currently married, and those with more children were less likely to favor it, compared to their respective counterparts. Additionally, the effect of education was stronger for liberals than for non-liberals. Race, family income, and full-time work status make no difference in the outcome. The findings have significant implications for research and practices in health insurance coverage for legal abortion.

## 1. Introduction

The debate about restriction to legal abortion access is again taking a center stage in the United States. Germane to this debate, whether it is apropos to use health insurance to cover legal abortion is also a controversial issue facing American society today. Currently, there is little quantitative research on this issue to inform policy and practices because of a lack of data. It is important to understand where the American public stands on this issue, so we know what should be done next. It is also crucial to fathom who is more or less likely to support the use of health insurance for legal abortion so all sides of the debate know who to ally with, who to win over, and who to fight with in order to enact appropriate legislation and bring about social change.

One of the dilemmas women tend to encounter is the decision to have or not to have an abortion. Despite many social advances in feminism, abortion reforms and discussions are labelled as highly controversial in many parts of the United States [1]. Women’s rights have been rallied for decades, yet the idea for a woman to receive an abortion remains stigmatized and hushed. As a society, we have constructed women’s motherhood role, and it has become almost expected [2]. On the other hand, the right to abortion is also socially constructed. A denial of either the social construction of motherhood or the social construction of the right to abortion is overly simplistic and neglects the dual character of human nature. Currently, most research tends to focus on the policy of abortion, moral rights, and the mental health of women receiving an abortion, but not much about health insurance coverage for an abortion [1,3,4,5].

However, some state policies allow for abortion [1,3], but heavily regulate the procedure; thus, a new question concerning if women seeking a legal abortion should be able to use health insurance has arisen in recent years. In this paper, we focus on the research question of who is more or less likely to support using health insurance for legal abortion. We address this question using the latest data from General Social Survey (GSS) collected in 2018. In the remainder of this paper, we review the historical background and current status of abortion and funding, propose hypotheses for testing, depict our data and methods, and present and discuss our findings.

### 1.1. Historical Background and Current Status

To understand the significance of legalizing abortion, one must first understand the history of abortion in the United States. The topic of abortion has had a long-disputed history in the U.S., which has witnessed the development of many of the policies in today’s society.

Planned Parenthood provides a list of procedures and expectations for performing an abortion in their clinics [6]. The procedure itself only takes around five to ten minutes, and they provide onsite counseling, examination, and medication to ease any pain during and after the procedure [6]. Currently, the United States consistently allows an abortion before twenty weeks, but with extenuating circumstance a late term abortion is permissible when the fetus is nearly fully developed [6]. The first step consists of an ultrasound to confirm that the woman is within the legal time frame to receive an abortion.

Once the ultrasound is completed, the health care provider will provide information to determine which form of abortion will be performed. The most common is the suction abortion [6]. The health professional will administer an over-the-counter pain medication to help with cramping, a sedation to keep the woman relaxed, and an antibiotic to help in preventing an infection [6]. Next, after numbing and widening the cervix, a small tube is inserted with a hand-held suction machine to remove any pregnancy tissue.

However, the organization also states they perform a dilation and evacuation procedure, which is the method typically used after sixteen weeks. This form of abortion usually takes a couple of days versus just the five-to-ten-minute procedure. To open the cervix for the procedure, the health care professional will insert a laminaria to help absorb the fluid from the body, and widen the cervix [6]. Just like in the suction procedure, the woman is issued over-the-counter pain medications for cramping, a sedative, and antibiotics to prevent infection [6]. From here, the same steps are taken as the suction abortion.

These are the currently allowed procedures in most of the United States. However, the question is: how did we as a society get our opinions in which most hold today about abortion? The first recorded abortion occurred in colonial America, and was a practiced, legal procedure [7]. According to Dine [7]), the procedure was common enough that it was added through legal and medical records. In fact, abortion helped boom early pharmaceutical companies for drugs to help induce abortion [8,9]. Newspapers raved about abortion medication, mailed flyers were used, and it was even advertised that if the home remedy did not work, a practitioner would be able to perform “instrumental procedures” [8,9]. At the time, the only attempt to govern the abortion was to reduce the poisoning side effects from the home medications [8,9].

However, the politicization of abortion began in the late 1800s [8,9]. When the term “quickening” (i.e., feeling the fetus moving) became widely used, then the medical establishments urged the banning of abortion unless deemed necessary to save a woman’s life [8,9]. By the early 1900s, abortion had been related to female independence, threatening male dominance [7]. One of the biggest pushes for the illegalization of abortion and the abortion drugs was led by OB-GYN Dr. Horatio Storer [8,9]. Many believe his opposing rally against abortion was centered around females aspiring to become doctors at Harvard Medical School where he devoted much of his medical practice [9]. He virtually succeeded criminalizing abortion and abortion drugs in the majority of the United States. Other opponents to abortion included female physicians such as Elizabeth Blackwell and Charlotte Lozier, who both believed abortions were sinful and dangerous [10]. It is also worth mentioning that many of the early leaders of the U.S. women’s suffrage movement (e.g., Susan B. Anthony, Elizabeth Cady Stanton, Victoria Woodhull, Elizabeth Blackwell) deemed abortion as “infanticide” [10]. They believed that the rights of mother and child are intricately tied and that the right to life and the right to vote are rooted in the inherent dignity of each human person. However, they were skeptical about the criminalization of abortion [10].

Despite the illegalization of abortion, many continued to have underground, dangerous, life-threatening procedures [7,8,9]. One of the physicians performing abortions behind closed doors stated that the majority of those receiving an abortion were middle to upper class, Protestant, married women [8,9]. However, it was not until the 1973 Supreme Court Case *Roe* vs. *Wade* that abortion once again became legal in the United States [7,8,9]. Nevertheless, in 1977, the Hyde Amendment prohibited the use of federal funds for an abortion unless the pregnancy was determined to be the result of “rape, incest, or if it is determined to endanger the woman’s life” [11].

Currently, abortion is legal in every state; however, there is a push to overturn the *Roe* vs. *Wade* decision [12]. With repeated failures at the national level, anti-abortion forces have shifted the focus to the state level. As a result, many laws have been enacted at the state level, heavily regulating and restricting the access to abortion [12]. Despite the heavy regulation, there are still some states that allow for health insurance coverage of legal abortion. The median cost for an out-of-pocket abortion between ten and twenty weeks is between USD 500 and USD 1200 [11]. As previously mentioned, the Hyde Amendment banned the use of federal funds, such as Medicaid, to help cover the costs of legal abortion. Around two-thirds of American women use Medicaid to help cover the costs of reproductive health, and many of these women are of lower income status [11]. One of the provisions for federally funded abortion is that the state can opt to cover all or part of an abortion [11]. However, less than twenty states choose to help cover the cost.

Private insurances are also heavily regulated by the state, and the majority choose the same provisions as federal programs, or have even stricter regulations for health insurance coverage for abortion [11]. Only California, Oregon, New York, and Washington State require private plans to have coverage for abortion with none or very light regulations [11].

### 1.2. Hypotheses

Since our research question is quite new, there is not much direct empirical evidence from the existing literature that can be used to shore up our hypotheses. Thus, we largely rely on theorization and some indirect evidence. We propose a number of hypotheses for testing. Survey data show women tend to be on the side of pro-choice on the abortion issue compared to men [13,14]. Since women are the ones who undergo abortion and may experience physical suffering, mental stress, and financial difficulty, they will benefit from health insurance coverage for an abortion. Thus, we hypothesize that, *all else being equal, women are more likely than men to support the use of health insurance to cover an abortion* for the interest of themselves and their families. Evidence indicates that younger people are more likely to support legal abortion than older people, but direct evidence between age and support for abortion health insurance coverage does not exist [14]. We anticipate *an inverse relationship between age and the likelihood of support for using health insurance to help with the costs of legal abortion, holding other variables constant*, because older people are generally more conservative than younger ones. Earlier studies of abortion attitudes found that blacks were less likely than whites to support legal abortion [15,16]. Furthermore, traditionally abortions were more likely to occur among white women than among black or other minority women. As of 2016, the overall rate of abortion based on race in the United States was highest among whites than among blacks or other races [17]. This suggests whites will benefit more from health insurance coverage for abortion than minorities. Hence, we expect that *racial minorities are less likely than whites to support health insurance coverage for abortion*, *ceteris paribus*.

The South is the hub of anti-abortion fervor [18]. Given the conservative tradition and environment in the South, we predict that *Southerners are less likely than residents in other regions to support health insurance coverage for legal abortion, controlling for other variables*. In the United States, urban areas tend to be more liberal than rural areas. Hence, we hypothesize that *urban residents are more likely than rural residents to favor the use of health insurance to cover legal abortion, other things being equal*. 

Religion was generally considered the strongest predictor of abortion attitudes [19]. In the U.S., Christians are the religious majority and tend to be more conservative. A large majority of Evangelical Protestants oppose legal abortion [14,19,20]. Christians also include Catholics whose religious tenets prohibit an abortion [19]. Orthodox Christians also condemn abortion. Despite some variation among Christian groups, Christianity advocates the “sanctity of human life” and generally opposes abortion more than other religions and the religious unaffiliated. Thus, we expect *Christians to be less likely than non-Christians to support using health insurance to cover legal abortion, holding other variables constant*.

Marriage and family can impact support for health insurance coverage for legal abortion. Available empirical evidence indicates that marriage reduces the likelihood of support for abortion [20]. Compared to their unmarried counterparts, married individuals are much less likely to opt for an abortion if pregnancy occurs. The need to cover an abortion is relatively lower for married people. Thus, we predict that *individuals who are currently married are less likely than those who are not currently married to support using health insurance to cover the cost of legal abortion*. Individuals who desire a larger family or have more children are less likely to need an abortion. Hence, we hypothesize *a negative relationship between the number of children and the probability to support health insurance coverage for legal abortion*.

Socioeconomic status can also influence support for the use of health insurance to cover legal abortion. Education makes people more open-minded [21,22]. Many studies have documented that more educated people are more likely to support legal abortion than less educated ones [23,24,25]. By extension, we hypothesize that *education is positively associated with the probability to support health insurance coverage for legal abortion*. Some prior studies reveal links between employment status and abortion attitudes [24,26]. A full-time job is normally associated with health insurance. Hence, it is reasonable to expect *individuals who hold a full-time job to be more likely than individuals who do not have a full-time job to support health insurance coverage for legal abortion* because they can benefit from their job-related insurance to cover an abortion should it happen. Family income means financial ability. Some evidence indicates an inverse association between income and support for abortion [24,27]. As an extension, we anticipate that *individuals with a higher family income are less likely to support health insurance coverage for abortion than those with a low family income* because they are more likely to be able to afford it if needed.

Abortion is a political issue, so political factors matter. It is well known that Democrats are generally more supportive of pro-choice than other parties [14,28,29,30,31]. Thus, we predict that to be consistent with their pro-choice stance, *Democrats are more likely to support health insurance coverage for abortion than non-Democrats*. It is also common knowledge that liberals tend to be pro-choice [14,29,30]. As an extension, liberals should be more supportive of health insurance coverage for abortion than non-liberals. Hence, we hypothesize that *liberals are more likely than non-liberals to support health insurance coverage for abortion*. We found that the correlation between the dummy variable for Democrat and the dummy variable for Liberal in our sample is 0.365. Thus, multicollinearity is not a concern, and the independent effects of both party affiliation and political ideology can be assessed simultaneously.

## 2. Materials and Methods

For this study, we used the GSS conducted in 2018 [32]. The GSS has surveyed non-institutionalized U.S. adult population aged 18 or older since 1972, covering demographic, behavioral, and attitudinal topics along with special topics of interest. We used GSS 2018 not only because it is the latest available GSS data but more importantly because the 2018 survey added for the first time the following new survey question: “People use their health insurance to help cover the cost of receiving health care. Do you think people should be able to use their health insurance to help cover the cost of receiving an abortion”? This new question provides the necessary information for us to study the current topic and to contribute to the continuous debate over abortion in the United States.

GSS 2018 is a full probability sample based on a multistage probability sampling design. Since the GSS only selected one respondent per household for survey, respondents in a larger household had a smaller chance of being selected than those in a smaller household. To address this bias, we used the weight variable designed by the GSS to weight the data, so that the findings can be generalized to the U.S. adult population. Our GSS sample statistics are similar to the available estimates of the U.S. population characteristics from the 2018 American Community Survey (ACS) collected by the U.S. Census Bureau. For example, in terms of gender composition, our GSS 2018 showed 53% female and 47% male for the U.S. adult population as compared to the estimates of ACS 2018 at 50.8% female and 49.2% male for the entire U.S. population. In terms of race, our GSS 2018 U.S. adult sample recorded 72.2% white, 15.1% black, and 12.7% other race, in comparison with 72.2% white, 12.7% black, and 15.1% other races combined (including 3.4% for two or more races) estimated by ACS 2018. GSS 2018 contained 49% of the currently married, which was similar to 47.8% of the currently married in ACS 2018. We restricted the analysis to the valid cases of the dependent variable on the use of health insurance to cover the cost of an abortion. The restricted sample contains 2,134 cases.

Table 1 provides the descriptions of the variables and their measurements used in the study. Since they are straightforward, only brief necessary notes are presented in order to conserve space. The dichotomous dependent variable measures whether the respondent supports or opposes using health insurance to cover legal abortion. Our independent variables include demographic characteristics (i.e., gender, age, race, region, urban/rural residency, religion, marital status, and number of children), socioeconomic status (i.e., education, employment, and family income), and political variables (i.e., political party affiliation, and political ideology). Family income is inflation-adjusted and converted to the 2000 constant U.S. dollar.

We first performed crosstabulations and χ^2^ tests for each predictor and the dependent variable. Since our dependent variable is dichotomous with many predictors, binary logistic regression is most appropriate to determine who is more or less likely to support using health insurance for legal abortion. The model takes on the form:

$$\ln\left(\frac{p_{i}}{1-p}\right)=a+\sum Bi_{}\;X_{i}$$

where ln$\left(\frac{p_{i}}{1-p}\right)$ is the logged odds ratio of supporting the use of health insurance to help cover legal abortion, *a* denotes the intercept, *B_i_* is the logistic coefficient for variables *X_i_* and *X_i_* represents the independent variables in the analysis. Some advantages of using a logistic regression model compared to a χ^2^ test include that multiple predictors can be included and explanatory variables can be discrete or continuous [33].

## 3. Results

### 3.1. Descriptive and Bivariate Analysis

Table 1 also displays the means and standard deviations of the variables used in the analysis. For a dummy coded variable, the mean can be interpreted as a percentage by multiplying the value by 100. Based on our sample statistics evident in Table 1, in 2018, roughly 50% (49.8% to be precise) of the Americans supported the use of health insurance to cover a legal abortion while the other half (50.2% to be exact) were against it. Note that our sample included 53% of women and 47% of men. Our sample comprised 15% blacks and 13% other race, versus 72% whites. On average, the respondents were about 46 years old with a standard deviation of 17.8 years. Almost two out of five resided in the South. Nearly 90% were urban dwellers. A large majority (71%) of the respondents were Christian versus 29% non-Christian. Nearly half were currently married. On average, the respondents reported almost 2 children. They also reported an average of 13.7 years of schooling, which equated to some college. Half of the respondents held a full-time job. On average, the respondents reported an annual family income of approximately USD 54,231 in the 2000 constant dollars. The sample was composed of 30% Democrats versus 70% non-Democrats, and 30% liberals versus 70% non-liberals. 

To gain a further understanding of the relationships between the predictors and support for using health insurance to cover legal abortion, we cross-tabulated each predictor and the dependent variable and conducted chi-squared tests. For the feasibility of these analyses, we collapsed several continuous variables including age, education, number of children, and family income. The results are shown in Table 2. Except for age, race, employment status, and family income, all other predictors display a significant relationship with the dependent variable because the χ^2^ values are significant at least at the 0.05 level. Among the significant predictors, most relationships are congruent with our hypotheses. One exception is gender, as surprisingly women (47.6%) were somewhat less likely to favor the use of health insurance for abortion than men (52.2%). However, the results of these bivariate analyses are tentative because other factors that could affect the dependent variable have not been controlled. In order to ascertain the true relationships, multivariate analysis is called for.

### 3.2. Multivariate Analysis

Table 3 presents the results of the two logistic regression models. Model 1 is the full model including all predictors. Model 2 adds the interaction term Education x Liberal to Model 1.

The model fit statistics indicate that Model 1 fits the data very well as indicated by the highly significant model χ^2^ (=0.356). The pseudo R^2^ indicates that Model 1 explains 23.3% of the variation in the probability of support for using health insurance for legal abortion.

The parameter estimates in Model 1 represent the independent effects of the predictors on the dependent variable. As shown in Model 1, except for race all demographic variables have a significant effect on support for the use of health insurance for legal abortion. The logistic regression coefficient for the female dummy variable is significant at the 0.05 level, but the sign is in the unexpected opposite direction. The odds ratio (=0.806) indicates that women were 19.4% (=0.806 − 1 = −0.194) less likely than men to support the use of health insurance for legal abortion. These results are at odds with our hypothesis. To test the possibility that an inadequate control of political orientation may have impacted the gender difference in support for the use of health insurance for abortion, we created another dummy “middle of the road” for political orientation, used conservative as the reference category, and reran the model. The result of a greater propensity of men over women in favor of insurance coverage for abortion remains unchanged. In addition, we created two interaction terms: Female x Liberal, and Female x Democrat and reran the model separately one at a time for each of the two interaction terms. Both interaction terms are not significant at the 0.05 level. However, among both liberals and non-liberals, women were still less likely than men to support health insurance for abortion; this was also true among Democrats and non-Democrats. Thus, women’s lower propensity than men to favor health insurance for legal abortion is not very likely to be a statistical artifact.

Age is also significant at the 0.05 level, but the effect contradicts our expectation since both the B and odds ratio show older people were more likely to support the use of health insurance for legal abortion. The dummy variable South is highly significant at the 0.001 level. As hypothesized, Southerners were about 33% (=0.674 − 1 = −0.326) less likely than residents in other regions to support the use of health insurance coverage for legal abortion. Additionally, as expected, urban residents were 1.48 times as likely as rural residents to support the use of health insurance for legal abortion.

Coinciding with our hypothesis, Christians were about 53% (0.475 − 1 = −0.525) less likely to support the use of health insurance coverage for legal abortion. We understand Christians encompass various groups. To test the differences among various Christian groups in support for the use of health insurance for abortion, we replaced the Christian dummy variable by three dummy variables for Christian groups with non-Christians as the reference category: Protestant, Catholic, and other Christian (Note: Since the number of Orthodox Christians was too small, we had to lump Orthodox Christian into the other Christian category), and we then reran Model 1. The results show that each of these three Christian groups was less likely than non-Christians to support health insurance for abortion with trivial changes in the effects of other predictors. Because of these results, we decided to merge all Christian groups into one category Christian for the efficiency of analysis and presentation.

Consistent with our hypothesis, currently married people were significantly less likely to support the use of health insurance to cover legal abortion than their not currently married counterparts. The effect of the number of children is also expected and significant at the 0.05 level. For each additional child, the odds of supporting the use of health insurance for abortion were predicted to decrease by 8% (0.920 − 1 = −0.08), holding all other variables constant. There were no significant differences between racial minorities and whites in support for the use of health insurance to cover legal abortion.

The effects of socioeconomic variables are mixed. As hypothesized, education has a highly significant positive effect on the dependent variable. For each additional year of schooling, the odds of supporting the use of health insurance for legal abortion were predicted to increase by 11.5% (1.115 − 1 = 0.115). However, full-time employment and family income had no significant effect on support for the use of health insurance to cover abortion.

Coinciding with common sense and our hypotheses, both party affiliation and political ideology are proven to be highly significant predictors of support for the use of health insurance to cover legal abortion. Significant at the 0.001 level, Democrats were twice as likely as non-Democrats to support the use of health insurance to cover legal abortion. We reran Model 1 by replacing the Democrat dummy variable by three dummy variables for Republican, Independent, and other party. The results confirmed that Republicans, Independents, and other party were all less likely than Democrats (the reference category) to support health insurance for abortion. Because of these results, we decided to keep one dummy variable for Democrat for the efficiency of analysis and presentation. Similarly, as shown by the odds ratio in Model 1 liberals were 2.164 times as likely as non-liberals to support the use of health insurance for abortion.

Are effects of certain predictors (e.g., education, party affiliation) moderated by other predictors (e.g., political ideology, gender, religion, region)? To address this question, we created many cross-product terms to test the possible interaction or moderating effects. None of the interaction effects were significant at the 0.05 level, except for the interaction between education and being liberal. The results are presented in Model 2 of Table 3. Compared to Model 1, Model 2 fits the data significantly better with a significantly smaller −2 log likelihood, a significantly greater model χ^2^ (=0.361), and a significantly greater pseudo R^2^ (=0.236) with one additional degree of freedom. The interaction term Education x Liberal is significant at the 0.05 level. The B indicates that the effect of education on support for the use of health insurance to cover abortion is greater for liberals than for non-liberals.

## 4. Discussion

Our findings have significant implications for research on this issue and for practices. Our findings confirm the conventional wisdom and our hypotheses regarding the effects of political party affiliation and political ideology on support for health insurance coverage for abortion [14,29,30,31,32]. They suggest that political divides serve as the most important considerations for coalition, operation, or opposition in dealing with the abortion health insurance issue. Earlier studies of abort attitudes [24,28] only found weak associations between political variables and abortion attitudes. Perhaps time is different now as the battles for legal abortion have intensified and partisan and ideological divides have deepened in more recent years.

Our result of a significant positive relationship between education and the dependent variable is consistent with the findings about the relationship between education and abortion attitudes in the literature [23,27]. It suggests that educational attainment will help increase support for the use of health insurance to cover abortion [23,24,25]. The significant interaction effect between education and political ideology implies that the more educated liberals tend to be the strongest advocates for abortion health insurance coverage.

The result that women are significantly less likely to support health insurance coverage for abortion than men before and after controlling for other factors seems to be a conundrum but may have a reason. Past studies of abortion attitudes generate mixed results about the gender difference. While some studies [23,34] found men were more likely to support abortion than women, Legge’s research [27] detected the opposite to some extent after holding other predictors constant. Of course, our dependent variable is not the same as theirs but these dependent variables all pertain to support for legal abortion or the use of insurance to cover legal abortion. One probable explanation offered by Blake and Del Pinal dubbed the “motherhood hypothesis” postulates that women place more importance on motherhood than on reproductive freedom than men [35]. This proposition may also help explain the gender difference in support for health insurance coverage for abortion. This result also suggests that one cannot assume women will automatically support health insurance coverage for abortion and that men can also support abortion insurance coverage.

Legge found that older people were less likely to be associated with support for abortion [27], but Baker et al.’s earlier study [34] concluded that age is not a particularly strong predictor of abortion attitudes. Nevertheless, our finding about a positive relationship between age and support for health insurance coverage for abortion is not in line with the findings of both studies and challenges the conventional expectation. This result suggests that older people may be a group to win over for support for abortion insurance coverage.

Prior research points to the most preponderant role of religion in shaping abortion attitudes [19,27,35]. The finding in our study suggests that, albeit not most important, religion remains a very important determinant of attitudes toward abortion insurance coverage as it impacts nearly every aspect and moral decision [14,19,36]. Opposition to the use of health insurance coverage for abortion can be expected to hail from the bulk of the Christian groups. The South is another base of opposition. Resistance to abortion insurance coverage can also come from those who are married and have more children.

The data provided in this study are particularly important because as of 2021, some southern states now prevent abortions as soon as the doctors are able to find a fetal heartbeat [37]. Often, this is before the woman even starts experiencing pregnancy symptoms, leading to finding out about the pregnancy past the allotted abortion time frame. Women who are impregnated as a result of rape and incest are no longer excluded from abortion laws. It is imperative to understand that many parties are involved on both sides of the debate. The battle over abortion and its coverage by health insurance is not only about civil rights and women’s rights but also about profit as medicalization of abortion is a huge business industry. Consequently, many parties have a stake in medicalization of abortion and its coverage by one’s health insurance, including physicians, birth control clinics, pharmaceutical companies, and the health insurance industry [38].

## 5. Conclusions

There is a dearth of quantitative data on support for the use of health insurance to access abortion, instead of paying out of pocket for the procedure. To provide policymakers and practitioners with useful information on this issue, this study examines American attitudes toward support for health insurance coverage for abortion, using the latest new data from GSS 2018. The results show that the support and opposition were about evenly divided. The findings from the logistic regression analysis reveal that, holding other variables constant, Democrats, liberals, urban residents, the more educated, and the older were more likely to support health insurance coverage for legal abortion while women, Southerners, Christians, the currently married, and those with more children were less likely to favor it, compared to their respective counterparts. Additionally, the effect of education was stronger for liberals than for non-liberals. Race, family income, and full-time work status make no difference in the outcome.

Health insurance coverage for abortion is emerging as a critical issue that calls for additional research. Some of our findings, especially with regard to the effects of gender and age, will require verification from other data sources. Since our data are cross-sectional, longitudinal data will help capture the changing American attitudes toward this issue.

## Figures and Tables

**Table 1:** Description of Variables Used in the Analysis.

Variable	Measurement	Mean	Standard Deviation
*Dependent Variable*			
Support for health insurance to cover abortion	1 = Support, 0 = Oppose	0.498	0.50
*Independent variables*			
Gender	1 = Female, 0 = Male	0.530	0.499
Race			
Black	1 = Black, 0 = Else	0.151	0.358
Other	1 = Other, 0 = Else	0.127	0.333
Age	Years	46.302	17.755
Region	1 = South, 0 = Else	0.390	0.488
Urban	1 = Urban, 0 = Rural	0.890	0.310
Religion	1 = Christian, 0 = Else	0.710	0.452
Marital status	1 = Currently married, 0 = Else	0.490	0.500
# of Children	Number	1.820	1.677
Education	Years of schooling	13.720	2.989
Full-time job	1 = Full-time, 0 = Else	0.500	0.500
Family income	USD in constant 2000 U.S. dollars	54,230.75	43,971.079
Party affiliation	1 = Democrat, 0 = Else	0.300	0.459
Political ideology	1 = Liberal, 0 = Else	0.300	0.458

**Table 2:** Percentage Distributions of Support for Using Health Insurance Coverage for Legal Abortion by Predictors, GSS 2018.

Variable	Support for Health Insurance to Cover Abortion (%)	N	χ^2^
Gender		2134	4.458 *
Female	47.6		
Male	52.2		
Age		2134	3.941
18–29	53.6		
30–64	48.3		
65 or older	50.3		
Race		2134	5.542
White	48.9		
Black	55.8		
Other	47.8		
Region		2134	53.779 ***
South	56.1		
Non-South	39.8		
Urban/rural residency		2134	29.706 ***
Urban	51.8		
Rural	32.8		
Religion		2120	96.531 ***
Christian	42.9		
Not Christian	66.4		
Marital Status		2,134	40.046 ***
Currently married	42.7		
Not curr. married	56.4		
# of children		2130	15.681 ***
2 or less	52.5		
3 or more	43.1		
Education		2132	59.642 ***
College educated	56.6		
Not coll. educated	39.6		
Work Status		2132	0.604
Full-Time	50.6		
Not full time	48.9		
Family income		1940	2.722
Less than average	47.9		
Average or more	51.7		
Party affiliation		2106	94.114 ***
Democrat	65.8		
Non-Democrat	42.7		
Political ideology		2051	138.761 ***
Liberal	70.4		
Non-Liberal	42.0		

* *p* ≤ 0.05, ** *p* ≤ 0.01, *** *p* ≤ 0.001.

**Table 3:** Logistic Regression Estimates Predicting Support for the Use of Health Insurance to Cover Abortion, U.S. Adults, GSS 2018.

Predictor	Model 1		Model 2	
	B	Odds Ratio	B	Odds Ratio
Female	−0.215 * (0.105)	0.806	−0.215 * (0.0105)	0.807
Age	0.007 * (0.004)	1.007	0.007 * (0.004)	1.007
Race (Ref. = White)				
Black	0.079 (0.158)	1.082	0.082 (0.158)	1.085
Other	−0.117 (0.168)	0.889	−0.093 (0.169)	0.912
South	−0.395 *** (0.110)	0.674	−0.388 *** (0.110)	0.678
Urban	0.392 * (0.174)	1.480	0.378 * (0.174)	1.460
Christian	−0.0744 *** (0.112)	0.475	−0.725 *** (0.122)	0.484
Married	−0.573 *** (0.115)	0.564	−0.565 *** (0.115)	0.569
# of Children	−0.083 * (0.036)	0.920	−0.079 * (0.036)	0.924
Education	0.109 *** (0.020)	1.115	0.081 *** (0.024)	1.085
Full-time job	0.062 (0.110)	1.064	0.067 (0.110)	1.069
Family income in USD 1000	0.001 (0.001)	1.001	0.001 (0.001)	1.001
Democrat	0.727 *** (0.125)	2.069	0.698 *** (0.126)	2.010
Liberal	0.772 *** (0.124)	2.164	0.478 (0.580)	0.620
Education x Liberal			0.091 * (0.041)	1.095
Constant	−1.467 *** (0.359)	0.231	−1.097 (0.393)	0.334
−2 Log Likelihood	2218.904		2213.882	
Model χ^2^	356.048 ***		361.071 ***	
Pseudo R^2^	0.233		0.236	
df	14		15	
N	1895		1895	

* *p* ≤ 0.05, ** *p* ≤ 0.01, *** *p* ≤ 0.001. Notes: The odds ratio is the antilog of the B, and the standard errors are in parentheses.

## Data Availability

Publicly available data were used for this study. The data set can be found here: https://gss.norc.org/ accessed on 28 August 2020.
